# Associated Factors of Awareness and Knowledge About HIV/AIDS Among Women of Reproductive Age in Somaliland: Insights From a Nationwide Survey

**DOI:** 10.1155/arat/3425388

**Published:** 2025-03-26

**Authors:** Hodo Abdikarim, Yahye Hassan Muse, Abdisalam Hassan Muse

**Affiliations:** ^1^Faculty of Science and Humanities, School of Postgraduate Studies and Research (SPGSR), Amoud University, Borama 25263, Somalia; ^2^Research and Innovation Centre, Amoud University, Amoud Valley, Borama 25263, Somalia

**Keywords:** awareness, determinants, HIV/AIDS, interventions, knowledge, policies, public health, reproductive age, Somaliland, women

## Abstract

**Background:** Human immunodeficiency virus/acquired immune deficiency syndrome (HIV/AIDS) continues to be a major public health issue, particularly among women of reproductive age. This study was conducted to examine the factors that influence HIV awareness and knowledge among women in Somaliland.

**Methods:** The data used in this study were obtained from the Somaliland Demographic and Health Survey conducted between 2019 and 2020, which included a representative sample of women aged 15–49 years. Descriptive statistics and multivariable binary logistic regression analyses were performed to investigate the connections between various sociodemographic factors and HIV awareness and knowledge among the women.

**Results:** The investigation uncovered that the level of HIV awareness and knowledge among women in Somaliland was inadequate, with only a small percentage possessing accurate information about HIV transmission and prevention. The multivariate logistic regression analysis showed that the education level (AOR = 1.58, 95% CI: 1.23–2.03), age (AOR = 0.82, 95% CI: 0.71–0.94), marital status (AOR = 1.32, 95% CI: 1.09–1.61), and media exposure (AOR = 1.49, 95% CI: 1.19–1.87) were significantly linked to higher levels of HIV awareness and knowledge among women.

**Conclusions:** The research findings highlight the need for tailored interventions aimed at increasing HIV awareness and knowledge among women in Somaliland. To achieve this, it is essential to improve educational opportunities, conduct media campaigns, and eliminate cultural and socioeconomic obstacles that hinder the dissemination of precise information about HIV/AIDS. Collaboration between healthcare professionals, community leaders, and policymakers is vital for the development and implementation of successful interventions aimed at improving HIV awareness and knowledge among women in Somaliland.

## 1. Introduction

### 1.1. Background

Human immunodeficiency virus/acquired immune deficiency syndrome (HIV/AIDS) is one of the most pervasive pandemics across the globe, affecting over 38.4 million individuals worldwide, and 1.5 million people are newly infected with HIV/AIDS by the end of 2021 [1]. HIV can instantly impair a person's immune system, ultimately leading to AIDS, which is fatal and causes immune system malfunctions, making the individual susceptible to serious infections. As a result, HIV/AIDS poses a significant threat to a nation's human population [2].

Despite the fact that HIV infection is terminal, it can now be managed as a chronic illness, allowing those who have it to live long and healthy lives. This is due to increased access to effective HIV prevention, diagnosis, treatment, and care, including for opportunistic infections. Moreover, global HIV policies from organizations such as the World Health Organization (WHO), UNAIDS, and the Global Fund align with SDG target 3.3, which aims to eradicate the HIV epidemic by 2030 [3].

According to the WHO, HIV has resulted in 40.1 million fatalities by 2023, making it a significant public health concern worldwide. Despite advancements in the diagnosis, treatment, prevention, and understanding of HIV/AIDS, the number of deaths from HIV and its consequences continues to rise [4, 5]. In 2018, 770,000 individuals lost their lives to HIV-related causes, and 37.9% of the global population was living with the virus. Married women aged 15–44 years, who undergo more hormonal changes, microbial ecology, and physiological changes than men, accounted for the majority of the 24.5 million HIV cases receiving antiretroviral medication [3, 6–8]. Several factors are linked to the risk of contracting HIV, and the rate of new HIV infections is currently high. Previous studies have shown that several characteristics, including age, affluence, media exposure, drug and alcohol intake, and educational status, are linked to HIV infection [9–12].

Somalia has some of the world's poorest health indicators, ranking 13th in terms of overall health [13]. Over 3 decades of civil war and political instability have severely weakened Somalia's healthcare system, causing the displacement of more than 2.5 million people within the country [14, 15]. The country has been plagued by protracted conflicts, droughts, floods, and locust infestations that have resulted in sustained food crises, widespread poverty, and inadequate infrastructure [16]. It is alarming to note that Somalia has the highest maternal and infant mortality rates in sub-Saharan Africa, despite the region accounting for more than half of all maternal deaths worldwide [17]. According to UNAIDS data from 2021, there are approximately 7700 adults and children living with HIV in Somalia, with more than 45% of these cases being among women over the age of 15. Thankfully, the number of deaths related to HIV/AIDS in the country is relatively low, with less than 500 people dying from the disease in recent years [18].

In Somaliland, the first-ever demographic health survey revealed that 80% of women aged 15–49 were familiar with HIV/AIDS. However, 49% of these women expressed negative attitudes toward individuals living with HIV, and 64% reported avoiding purchasing fresh produce from a shopkeeper who was HIV-positive [19]. Despite the progress made in HIV/AIDS education and awareness, many reproductive-age women in developing countries, including Somalia, lack comprehensive knowledge about the disease [20]. Access to mass media, such as television, radio, and newspapers, has been shown to be an important factor in disseminating HIV knowledge and promoting awareness and education in communities [21].

To the best of our knowledge, there is a lack of research investigating these factors, specifically within the context of Somaliland. As a result, the objective of this study was to address this knowledge gap by investigating the knowledge of HIV among reproductive-age women in Somaliland and identifying demand-side factors that are associated with HIV in Somaliland. This can be achieved by utilizing nationally representative data, allowing for a comprehensive analysis of the topic at hand.

## 2. Review of the Literature

HIV/AIDS pose a significant challenge to global health, as over 70 million individuals have been infected, with 35 million losing their lives and 36.7 million currently living with the disease [22]. With a substantial amount of research conducted, HIV/AIDS has been the subject of more than 260,000 studies listed in PubMed [23] and over 42,000 papers in Web of Science [24], spanning 3 decades of scientific inquiry. The disease is investigated across a variety of fields, including epidemiology [25], virology [26], immunology [27], drug development [28], and social and humanity sciences [29, 30].

More than two-thirds of the global population of people living with HIV, which amounts to approximately 25.6 million individuals, reside in Africa [20]. The majority of HIV/AIDS patients are found in low-income and middle-income countries. In 2021, out of the 20.6 million individuals living with HIV in eastern and southern Africa, 5 million (13%) were located in western and central Africa [31]. Additionally, sub-Saharan Africa remains the region most severely affected by HIV, accounting for more than half of all HIV-positive individuals in the region and approximately 67% of the global HIV burden [32]. Some scholars argue that comprehensive knowledge about HIV/AIDS is not widely prevalent in Africa, particularly in sub-Saharan Africa, where the prevalence varies significantly, ranging from 19.3% in Ethiopia to 48.9% in Burundi [21, 33–38]. Many factors, including age, education, wealth status, place of residence, sex of household head, region, frequency of listening to a radio, frequency of watching television, and media exposure, are associated with comprehensive knowledge about HIV/AIDS [33, 39, 40].

A possible reason for the rising number of new HIV/AIDS infections is the widespread lack of comprehensive knowledge about the disease. Some academics argue that gaining a thorough understanding of HIV is crucial for curbing the epidemic [33–35]. Unfortunately, only 30% of women of reproductive age possess a comprehensive understanding of HIV/AIDS, even though they are at risk of contracting the virus globally [21].

The Somali HIV and AIDS epidemic, as reported in 2013, highlights the distribution of HIV-positive test results among TB patients in health facilities and those tested through voluntary counseling and testing. This finding further supports the observation that the epidemic is more concentrated in Somaliland and at low levels in South Central and Puntland [41]. Although there seems to be a decline in HIV prevalence among ANC attendees between 2004 and 2010/11, this reduction over time was not statistically significant. It has been suggested that the relatively higher HIV prevalence in Somaliland (which is three times higher than that in Puntland) may be attributed to substantial trade-driven mobility and interaction with neighboring countries, such as Ethiopia, which have higher HIV prevalence [37].

Despite the obstacles posed by accessibility, drought, and security issues, the WHO data show that by June 2022, a significant portion of patients receiving antiretroviral therapy (ART) were on newer, more effective dolutegravir (DTG)-based regimens, with 82.7% of the 3978 patients benefiting from this advanced treatment. The data further reveal that ART coverage for the estimated number of people living with HIV increased from 34.7% in 2018 to 44.7% in 2021. Furthermore, during 2021, 49.6% of all patients enrolled in ART for at least six months received an HIV viral load test, with 87.3% of these patients achieving virological suppression, meaning that the virus quantity had been reduced to a level where the treatment's effectiveness could be assessed. The percentage of patients achieving virological suppression increased from 69.1% in 2018, when the service was first introduced, and from 80.7% in 2020 [38].

Previous studies have identified various elements that could influence HIV rates. These elements comprise the educational level of mothers [40], exposure to media [42], and socioeconomic indicators, such as the wealth index [42] With the current gaps in knowledge, this study aimed to tackle this issue by investigating the HIV knowledge among women of reproductive age. Furthermore, the study intended to pinpoint the demand-side factors associated with HIV knowledge in Somaliland, employing nationally representative data.

## 3. Methods

### 3.1. Study Area

The present investigation was carried out in East African Somaliland, which is situated between the Gulf of Aden to the north, Ethiopia to the southwest, Djibouti to the northwest, and Somalia to the east. The nation spans an area of 176,119.2 km^2^ and features a mixed climate of dry and rainy conditions. The region is divided into five administrative regions: Awdal, Waqooyi-Galbeed, Togdheer, Sanaag, and Sool, which are home to both Muslims and members of the Somali ethnic group. With an estimated population of 4.2 million, the majority of residents in urban and rural areas rely on livestock products for their subsistence. Despite modest economic growth since its declaration of independence, Somaliland has continued to expand at a slow pace. The lack of international recognition as a sovereign state has limited foreign investment and assistance in the region [43–48].

### 3.2. Study Design

The Somaliland Demographic and Health Survey (SLDHS) conducted in 2019 employed a cross-sectional study design to gather comprehensive data on health and demographic indicators. This design allowed for the examination of various factors within the targeted population, resulting in valuable insights that can inform decision-making and address health-related challenges in Somaliland.

### 3.3. Sample Size and Sampling Technique

The current study used data from 6247 women aged 15–49 years, which were taken from the SLHDS dataset. Sampling considered residency (urban, rural, and nomadic) and five geographic regions for stratification. Geographic information system (GIS) software was employed to determine the enumeration area (EA) for both urban and rural areas. The sampling frame consisted of 2806 residential structures, with 1869 in urban areas and 937 in rural areas. A probability-based method was used to choose 35 EAs based on the size of residential structures. Following the listing of households in the 35 EAs, 10 primary sampling units (PSU) were chosen using the probability proportional sampling technique from among the 35 EAs. 24 temporary nomadic settlements (TNS) for itinerant residents were constructed with a sample frame in mind.

The projected number of households in each TNS served as a measure of size, and the list of TNS was used as the sampling frame. A total of 1448 TNS residential structures were found, and the EAs of both urban and rural inhabitants were chosen in the same manner. Systematic sampling approaches were used to select households as the final sampling units.

### 3.4. Variables

#### 3.4.1. Outcome Variable

This study aims to assess the level of understanding and familiarity with HIV/AIDS among women of childbearing age in Somaliland. The outcome variable for this analysis is a binary variable directly obtained from the SLDHS 2020 dataset. This variable is in the IR file and has a code of V751, indicating whether a respondent has ever heard of HIV/AIDS. Respondents who answered “Yes” to the question “Have you ever heard of AIDS or Acquired Immune Deficiency Syndrome?” were coded as 1, while those who answered “No” were coded as 0. This variable serves as the primary dependent variable in our multivariable binary logistic regression analysis.

#### 3.4.2. Independent Variables

Previous research has pinpointed several elements related to HIV/AIDS awareness and knowledge [17, 31–33]. This study categorizes the correlates of HIV/AIDS awareness and knowledge into two groups: individual-level factors and community-level factors. The individual-level factors include the maternal age group and the maternal education level, while the community-level factors encompass residence, region, household wealth status, frequency of listening to the radio, and frequency of watching television.

### 3.5. Data Source

The SLDHS supplied the information analyzed in this research (SLDHS). In the survey, trained interviewers employed the CSPro Android platform to collect data from both urban and rural areas. Thirty households in each of the 10 enumeration regions within each geographic stratum contributed data. In the same manner, information was collected from 30 randomly selected homes within each enumeration region in the nomadic regions. To guarantee that the list of homes was accurate and complete, it was cross-checked one day prior to the data collection day in each TNS.

### 3.6. Data Quality Assurance

Data collectors received training and took a pretest before collecting the survey data. Data collection was continuously monitored, and GPS tracking of field operations was used to assist with georeferencing so that geolocated data could be collected.

### 3.7. Data Processing and Analysis

The data were obtained from the SLDHS and underwent cleaning. Individuals who had no response variables in the datasets were eliminated from the analysis process. The data were then exported and analyzed using the STATA 17software. Descriptive statistics, including measures of frequencies and percentages, were determined. To examine the associated variables of awareness and knowledge of HIV/AIDS, a bivariable (chi-square test) and multivariable binary logistic regression analysis was performed.

## 4. Results

### 4.1. Characteristics of the Participants

Table 1 in the study presents the results of the univariate analysis of HIV/AIDS knowledge and awareness among women in Somaliland, using data from the 2020 SLDHS. The findings revealed crucial insights. Notably, the study indicated a considerable proportion of respondents in the 15–19 age group, accounting for 26.33% of the surveyed population, emphasizing their youthfulness. Moreover, educational attainment played a vital role, with an alarming majority (72.72%) reporting no formal education, which could impede access to essential information. The study also revealed limited exposure to mass media, particularly radio and television, with a significant majority of respondents reporting no exposure (94.00% for radio and 81.42% for television), suggesting gaps in information-dissemination strategies.

Regional differences in HIV awareness and knowledge exist, as shown by the higher proportions in Marodijeh, Togdheer, and Sanaag regions compared to Awdal. Consequently, targeted interventions are necessary. Moreover, awareness and knowledge differ based on the type of residence, with urban areas demonstrating higher levels than rural and nomadic settings. This highlights the significance of accessibility to healthcare and information resources. Additionally, the wealth index plays a substantial role, with higher wealth categories correlating to better understanding. This indicates the impact of socioeconomic disparities on HIV awareness and knowledge.

### 4.2. Prevalence of the Awareness and Knowledge About HIV/AIDS

Table 2 presents the prevalence of awareness and knowledge about HIV/AIDS among women of reproductive age (15–49 years) in Somaliland. The table displays the proportions and corresponding standard errors, as well as the 95% confidence intervals for the two categories: “Yes” and “No.” Among the women surveyed, approximately 73.7% (with a standard error of 0.0055703) reported that they had heard of HIV/AIDS at some point in their lives. The 95% confidence interval suggests that the true proportion of women who have heard of HIV/AIDS lies between 72.6% and 74.8%. On the other hand, around 26.3% (with a standard error of 0.0055703) of the women indicated that they had not heard of HIV/AIDS. The 95% confidence interval indicates that the true proportion of women who have not heard of HIV/AIDS falls between 25.2% and 27.4%. These findings provide insights into the awareness level regarding HIV/AIDS among women of reproductive age in Somaliland, highlighting that a significant majority of surveyed women have heard of HIV/AIDS, while a notable proportion remain unaware of it.

### 4.3. Analysis of Associations

Table 3 in the study analyzes the bivariate analysis of HIV/AIDS awareness and knowledge among women in Somaliland, based on the 2020 SLDHS data. The results indicate several significant associations, highlighting that younger age groups, lower education levels, limited exposure to mass media, rural and nomadic residences, and lower socioeconomic status are all associated with decreased levels of HIV/AIDS awareness and knowledge. On the other hand, older age, higher education levels, more frequent exposure to mass media, urban residences, and higher socioeconomic status are correlated with increased awareness and knowledge levels. These findings stress the importance of designing targeted interventions that cater to specific demographic and socioeconomic contexts to enhance HIV/AIDS awareness and knowledge among women in Somaliland, ultimately contributing to more effective public health strategies for combating the spread of the disease.

### 4.4. Model Diagnostics

Prior to conducting logistic regression, we assessed for multicollinearity among the independent variables. This was performed using the variance inflation factor (VIF). The VIF values for each independent variable are presented in Table 4. The mean VIF for all variables was 1.67. Using a VIF threshold of 5 as an indicator of problematic multicollinearity and given the highest VIF was 2.93, we concluded that multicollinearity was not a significant concern. Therefore, no variables were removed or adjusted due to multicollinearity.

### 4.5. Multivariable Logistic Regression Analysis

Table 5 provides the results of a multivariate logistic regression analysis that explores the factors connected with the knowledge and awareness of HIV/AIDS among women in Somaliland using data from the SLDHS, which was carried out in 2020. The analysis uncovered several significant associations.

Age was found to be a significant predictor of awareness and knowledge of HIV/AIDS. Women in all age groups, except for the reference group (15–19 years old), had higher odds of having awareness and knowledge. For example, women in the 20–24 age group exhibited 0.554 times lower odds of awareness and knowledge than the reference group. Similar patterns were observed for the other age groups (25–29, 30–34, 35–39, 40–44, and 45–49), indicating lower odds of awareness and knowledge. The education level was positively associated with awareness and knowledge of HIV/AIDS. Women with primary, secondary, and higher education levels had significantly higher odds of awareness and knowledge than those with no education. The odds decreased with increasing education levels, suggesting a gradient effect.

The lack of a statistically significant relationship between radio listening frequency and HIV/AIDS awareness and knowledge was demonstrated. Individuals in the “less than once a week” and “not in all” categories did not differ significantly from the reference group (“at least once a week”). Correspondingly, television viewing frequency was not found to be significantly associated with HIV/AIDS awareness or knowledge. Nevertheless, the “not at all” category came close to reaching statistical significance (*p* = 0.084), suggesting a possible pattern.

Based on the information provided, it can be inferred that women from the Marodijeh, Togdheer, and Sanaag regions showed a higher likelihood of having knowledge and awareness about HIV/AIDS when compared to women from the Awdal region, which served as the reference group. Furthermore, the Sool region demonstrated a positive association, although it was not statistically significant. When examining the type of residence, women in urban and nomadic areas had significantly higher odds of possessing knowledge and awareness about HIV/AIDS when compared to women residing in rural areas, which served as the reference group. Specifically, women in urban areas had 1.47 times higher odds, while women in nomadic areas had 2.80 times higher odds of awareness and knowledge.

Additionally, there was no statistically significant relationship between the wealth index and awareness or knowledge of HIV/AIDS. All wealth index categories, including second, middle, fourth, and highest, did not show any significant differences when compared to the reference group (lowest).

## 5. Discussion

This study's results on the association between various factors and HIV/AIDS awareness among women align with and add to the existing body of the literature in this area. Previous research conducted in different settings has also explored the relationship between these factors and HIV/AIDS awareness, providing additional insights and supporting the current findings.

The study found a significant connection between age and HIV/AIDS awareness among women. Except for the reference group (15–19 age group), women in all other age groups had significantly higher odds of awareness and knowledge. Regarding age, the lower odds of awareness and knowledge among younger women align with studies conducted by Smith [49] in the United States and [50] in Sub-Saharan Africa. These studies found that older individuals generally have higher levels of HIV/AIDS knowledge due to greater exposure to HIV/AIDS education and prevention programs throughout their lives.

This research revealed a relationship between the education level and HIV/AIDS awareness or knowledge, demonstrating that women with primary, secondary, and higher education had higher odds of being knowledgeable about HIV/AIDS compared to those without any education. This finding aligns with studies conducted in various regions, such as China and Southeast Asia, which found similar results [51, 52]. Research conducted in these areas revealed that higher education levels were associated with greater HIV/AIDS knowledge among women.

In line with [53] findings in the United States, there is no substantial connection between radio and television exposure and HIV/AIDS awareness/knowledge. Their research revealed that media exposure alone did not make a substantial impact on HIV/AIDS knowledge among young adults. Therefore, additional approaches may be necessary to enhance awareness and knowledge levels effectively.

The lack of a statistically significant association between radio and television exposure and HIV awareness in our study is a noteworthy finding that requires further exploration. While these forms of media have the potential to reach large populations, several factors in Somaliland may explain the absence of a significant relationship. First, access to radio and television, especially in rural and nomadic areas, is often limited due to logistical challenges, availability of electricity, and cost of devices, leading to skewed reach among certain groups, as highlighted in the study data (94% of participants reported no radio access and 81% reported no TV access). Second, the content and format of existing HIV-related messaging may not be culturally appropriate, targeted, or trusted by the local population. If messages are not easily understood or perceived as disconnected from lived experiences, they are unlikely to influence knowledge or behavioral changes. Furthermore, it is also plausible that the frequency of relevant health-related messages is low, meaning that the participants are rarely exposed to such information even if they have access to media. Finally, even if the media exposure is available, the use of such media might not be for educational purposes and could be limited to other purposes, thus limiting the impact on health-related matters. Therefore, it is imperative to move beyond the simple provision of information through radio and television to a more comprehensive approach that considers targeted messaging, alternative media channels, trusted local community leaders as sources of information, and other contextual factors to enhance awareness.

The outcomes of the region-wise analysis revealed that women residing in specific regions, namely Marodijeh, Togdheer, and Sanaag, had significantly higher odds of being aware and knowledgeable about HIV/AIDS than those living in the Awdal region, which served as the reference group. Additionally, the Sool region demonstrated a positive association, although this association was not statistically significant. These findings illustrate the existence of regional disparities in HIV/AIDS awareness and knowledge, emphasizing the need for tailored interventions that address the unique needs of each region. Previous research conducted in diverse regions has also found variations in HIV/AIDS knowledge across different geographic areas. For instance, [54] conducted a study in multiple countries, including sub-Saharan Africa, and discovered differences in HIV/AIDS knowledge across various regions. Similarly, [55] conducted a study in Southern Africa and reported similar results, highlighting the impact of geographical context on HIV/AIDS awareness.

The present study's results on the connection between place of residence and HIV/AIDS awareness/knowledge correspond with the findings from various international studies. For instance, Mitiku and colleague [56] conducted research in Ethiopia and reported that urban residents demonstrated higher levels of HIV/AIDS knowledge than those living in rural areas. Similarly, Bintabara and colleague [57] conducted a study in Tanzania and found disparities in awareness levels between urban and rural populations.

The nomadic population of Somaliland presents a unique context that significantly influences their HIV awareness and knowledge. Their frequent mobility in search of pasture and water results in limited access to settled healthcare facilities and consistent health information. This, combined with lower rates of literacy due to limited access to formal education and reliance on traditional practices and community leaders as the primary source of health advice, increases their vulnerability to misinformation and reduces their access to health information. The logistical challenges of reaching nomadic communities also mean that they are less likely to benefit from standardized health outreach programs. These factors contribute to the significantly lower levels of HIV awareness found in this population compared to their urban counterparts, as highlighted in our study. Therefore, evidence-based interventions for nomadic populations must be tailored to their specific needs by providing regular mobile health clinics that can travel with the communities, working with trusted community leaders to disseminate health information, and employing health education approaches that do not rely on literacy but that is in line with their culture. A holistic approach is required to reach this uniquely vulnerable population, involving tailored outreach programs, culturally appropriate health information, and community involvement [44, 45, 58].

The lack of a statistically significant association between the wealth index and HIV/AIDS awareness/knowledge is consistent with the findings of Dinku in Ethiopia [59]. Specifically, their study explored the relationship between socioeconomic status and HIV/AIDS knowledge among women and did not reveal a significant association. This suggests that factors beyond wealth, such as education or access to healthcare, may play a more significant role in shaping awareness and knowledge levels.

In addition, the findings of this study revealed that while a majority (73.7%) of women in Somaliland had heard of HIV/AIDS, significant gaps in knowledge remain, particularly among those with lower education levels and in rural or nomadic areas. This uneven distribution of awareness is likely exacerbated by the pervasive stigma surrounding HIV within Somaliland. The documented negative attitudes toward individuals living with HIV, as noted in the introduction and supported by similar findings in other studies, likely deter women from seeking testing or openly discussing HIV within their communities, contributing to the lower levels of awareness. Specifically, the significantly lower odds of HIV awareness among women with no education (OR = 0.46, *p* = 0.000) and those in rural areas may be partially attributable to the fear of potential social exclusion and judgment. Furthermore, this fear of stigma can hinder open communication within families and communities, limiting access to accurate information and potentially contributing to the misconceptions surrounding HIV transmission and prevention that are prevalent in this region. Given that this study found higher levels of awareness among those with higher education and urban settings, these findings highlight the significant influence of societal barriers in the spread of HIV information, demonstrating that providing information alone is not enough, and there must be a conscious effort to combat stigma in order to effectively improve HIV awareness and knowledge.

Finally, while our findings on the influence of age, education, and residence on HIV awareness align with global trends, the specific context of Somaliland, marked by a history of conflict and resource constraints, significantly shapes these associations. The legacy of protracted conflict has severely weakened the healthcare infrastructure, limiting access to essential HIV prevention programs, particularly in rural and nomadic areas. Limited resources and a strained health system not only constrain consistent and impactful intervention but also impede general access to care, potentially leading to lower testing rates. Furthermore, years of instability have hindered educational opportunities for many women, especially in rural areas, thus affecting literacy rates and the ability to readily understand text-based HIV information. Traditional cultural norms can limit women's autonomy in seeking healthcare, impacting the uptake of testing and treatment services. These challenges are further exacerbated by a strong community focus where misinformation may spread if health information is not provided through trusted leaders or from health providers. The complexities of the post conflict setting, therefore, highlight the need for tailored, multifaceted interventions that acknowledge and address the challenges, aiming to create contextually relevant interventions, particularly for those vulnerable communities of nomadic settings, with limited education and rural residences.

### 5.1. Strength and Limitations

This study boasts several strengths, such as its valuable addition to the current literature on HIV/AIDS awareness and knowledge among women, a robust research design, and a comprehensive analysis of multiple factors. The study's confirmation and expansion of previous findings strengthen the overall understanding of the determinants of HIV/AIDS knowledge. The practical applications of this study can guide the development of targeted interventions and policies to enhance HIV/AIDS awareness and knowledge among women, ultimately benefiting public health efforts in this area.

One potential limitation of this study is its cross-sectional design. Cross-sectional studies only provide a snapshot of participants' awareness and knowledge at a specific point in time, which may limit the ability to draw causal conclusions. To overcome this limitation, longitudinal studies that allow for the examination of temporal associations between the factors studied and HIV/AIDS awareness and knowledge would provide more robust insights. However, the study relied on self-reported data, which can introduce potential limitations. Self-reporting is subject to recall and social desirability biases, where participants may overestimate or underestimate their actual knowledge levels. This could affect the accuracy and validity of the findings, and readers should exercise caution when interpreting the results.

The purpose of this statement is to emphasize that the study's design does not establish a causal relationship between the identified factors and HIV/AIDS awareness or knowledge. It is important to note that other unmeasured variables or confounding factors may have influenced the observed associations, and alternative explanations should be taken into account. The study's focus on a specific set of factors (age, education, media exposure, region, and place of residence) means that other potentially relevant factors, such as cultural beliefs, social norms, and access to healthcare services, were not considered. This omission limits the comprehensiveness of the analysis and may not provide a complete understanding of HIV/AIDS knowledge among women.

This study did not have a comparison group, such as men or different age groups. Including such groups could provide valuable insights into the sex- or age-specific differences in HIV/AIDS awareness and knowledge. It is important to note that the study may have been conducted several years ago, and since then, the HIV/AIDS landscape and interventions may have evolved. Therefore, it is crucial to consider the relevance and applicability of these findings to the current context.

Additionally, although this study controlled for certain variables, there may still be unmeasured confounding factors that were not accounted for. These unmeasured factors could potentially influence the observed associations and should be addressed in future studies. Acknowledging these limitations helps to recognize the potential weaknesses of the study and highlights areas for future research and improvement in study design and data collection methods.

Finally, our analysis is also limited by the absence of data on attitudes toward HIV/AIDS and people living with HIV. Negative attitudes, such as stigma and discrimination, can significantly impede HIV prevention efforts, even when awareness and knowledge are high. It is possible that negative attitudes may explain some of the observed disparities in HIV/AIDS-related behaviors. For example, even if a woman is aware of HIV transmission routes, she might avoid testing due to fear of social stigma if she tests positive. Future studies should consider incorporating validated scales to measure attitudes, such as those assessing stigma, fear, and social distance, to provide a more nuanced understanding of the factors influencing HIV/AIDS in Somaliland. This could involve using existing scales adapted for the local context or developing new scales that capture culturally specific attitudes.

## 6. Conclusion

In conclusion, this study highlights critical gaps in HIV awareness and knowledge among women in Somaliland, underscoring the need for targeted, context-specific interventions. Our findings indicate that while a majority of women have heard of HIV, knowledge is not equitable across demographic groups, with women in rural and nomadic areas, as well as those with lower levels of education, being most vulnerable. To address these disparities, several action-oriented steps are essential. First, for women with low education levels, community-based education programs utilizing participatory approaches and incorporating visual aids are essential, as this approach may address their low literacy levels. These programs should be delivered by trusted community members and local health workers and should be tailored to the needs of the local communities. Second, mobile health clinics and outreach programs should be implemented to address the unique needs of the nomadic population, ensuring access to HIV testing, counseling, and essential health information. These programs should work with community leaders and members of the community to implement them. Third, the utilization of culturally appropriate communication channels is key, moving beyond generic media campaigns to leverage trusted local leaders, community events, and storytelling, to convey HIV prevention and management messages to the wider population. Fourth, it is crucial to strengthen the healthcare infrastructure by training health professionals and integrating HIV prevention into general health programs, as well as providing adequate access to testing and treatment in the regions where it is limited. Finally, given that knowledge gaps might stem from misinformation driven by stigma, it is essential to work with local organizations to develop community-led antistigma campaigns and promote open communication about HIV. Addressing these challenges with context-specific and culturally appropriate interventions is vital for improving HIV awareness, knowledge, and ultimately, health outcomes for women in Somaliland.

### 6.1. Future Work

Future research can build on the findings of this study by exploring various avenues. Firstly, conducting longitudinal studies would provide valuable insights into the temporal dynamics of HIV/AIDS awareness and knowledge among women. By examining changes in awareness and knowledge over time, researchers can better understand the factors that contribute to improvements or declines in knowledge levels and identify strategies to sustain and enhance knowledge over the long term.

Incorporating qualitative research methods alongside quantitative findings can provide a deeper comprehension of the contextual factors that impact HIV/AIDS awareness and knowledge among women. Techniques such as in-depth interviews or focus groups enable an exploration of the women's lived experiences, viewpoints, and beliefs, thereby illuminating the social and cultural aspects that influence their knowledge levels. This approach offers a more refined understanding of the obstacles and facilitators of HIV/AIDS awareness and knowledge.

Another important area for future research is the design and implementation of targeted interventions to improve HIV/AIDS awareness and knowledge in women. Evaluating the effectiveness of educational programs, community awareness campaigns, and innovative interventions can inform best practices and help identify the most effective strategies for enhancing knowledge levels. Additionally, assessing the impact of improved knowledge on behaviors and health outcomes, such as preventive practices and health-seeking behaviors, can provide insights into the broader public health implications of increased awareness and knowledge.

Investigating the intersections of social identities, including race, ethnicity, and socioeconomic status, with HIV/AIDS awareness and knowledge among women is a crucial area for future research. Understanding how these factors intersect to shape knowledge levels can provide valuable insights into the underlying mechanisms and inform the development of more inclusive and targeted interventions.

Additionally, exploring the potential of technology and digital interventions, such as mobile health applications and online platforms, to improve HIV/AIDS awareness and knowledge among women is an emerging area of interest. Assessing the feasibility, acceptability, and effectiveness of these innovative approaches can provide valuable insights into their potential impacts and scalability.

## Figures and Tables

**Table 1:** Univariate analysis of awareness and knowledge of HIV/AIDS among women in Somaliland using SLDHS 2020 data.

Variable	Levels	Frequency (%)
Age in 5-year groups	15–19	1645 (26.33)
	20–24	1109 (17.75)
	25–29	1072 (17.16)
	30–34	876 (14.02)
	35–39	762 (12.20)
	40–44	486 (7.78)
	45–49	297 (4.75)

Respondent's highest education level	No education	4543 (72.72)
	Primary	1007 (16.12)
	Secondary	503 (8.05)
	Higher	194 (3.11)

Radio listening frequency	At least once a week	264 (4.23)
	Less than once a week	111 (1.78)
	Not at all	5872 (94.00)

Television watching frequency	At least once a week	930 (14.89)
	Less than once a week	231 (3.70)
	Not at all	5086 (81.42)

Region	Awdal	843 (13.49)
	Marodijeh	1360 (21.77)
	Togdheer	1434 (22.96)
	Sool	1244 (19.91)
	Sanaag	1366 (21.87)

Type of place of residence	Urban	2387 (38.21)
	Rural	1906 (30.51)
	Nomadic	1954 (31.28)

Wealth index	Lowest	1836 (29.39)
	Second	874 (13.99)
	Middle	712 (11.40)
	Fourth	1124 (17.99)
	Highest	1701 (27.23)

**Table 2:** Prevalence of the knowledge and awareness about HIV/AIDS among women of reproductive age (15–49 years) in Somaliland.

Ever head HIV/AIDS	Proportion	Standard error	[95% confidence interval]
Yes	0.74	0.01	(0.73, 0.75)
No	0.26	0.01	(0.25, 0.27)

**Table 3:** Bivariate analysis of knowledge and awareness of HIV/AIDS among women in Somaliland using SLDHS 2020 data.

Variable	Levels	Frequency (%)	Ever head of HIV/AIDS		Chi square	Df	*p* value
			Yes (%)	No (%)			
Age in 5-year groups	15–19	1645 (26.33)	1079 (65.59)	566 (34.41)	80.45	6	0.000
	20–24	1109 (17.75)	826 (74.48)	283 (25.52)			
	25–29	1072 (17.16)	818 (76.31)	254 (23.69)			
	30–34	876 (14.02)	677 (77.28)	199 (22.72)			
	35–39	762 (12.20)	593 (77.82)	169 (22.18)			
	40–44	486 (7.78)	375 (77.16)	111 (22.84)			
	45–49	297 (4.75)	236 (79.46)	61 (20.54)			

Respondent's highest education level	No education	4543 (72.72)	3098 (68.19)	1445 (31.81)	290.25	3	0.000
	Primary	1007 (16.12)	843 (83.71)	164 (16.29)			
	Secondary	503 (8.05)	471 (93.64)	32 (6.36)			
	Higher	194 (3.11)	192 (98.97)	2 (1.03)			

Radio listening frequency	At least once a week	264 (4.23)	244 (92.42)	20 (7.58)	60.67	2	0.000
	Less than once a week	111 (1.78)	96 (86.49)	15 (13.51)			
	Not at all	5872 (94.00)	4264 (72.62)	1608 (27.38)			

Television watching frequency	At least once a week	930 (14.89)	845 (90.86)	85 (9.14)	208.56	2	0.000
	Less than once a week	231 (3.70)	206 (89.18)	25 (10.82)			
	Not at all	5086 (81.42)	3553 (69.86)	1533 (30.14)			

Region	Awdal	843 (13.49)	618 (73.31)	225 (26.69)	208.87	4	0.000
	Marodijeh	1360 (21.77)	1171 (86.10)	189 (13.90)			
	Togdheer	1434 (22.96)	1052 (73.36)	382 (26.64)			
	Sool	1244 (19.91)	761 (61.17)	483 (38.83)			
	Sanaag	1366 (21.87)	1002 (73.35)	364 (26.65)			

Type of place of residence	Urban	2387 (38.21)	2061 (86.34)	326 (13.66)	573.42	2	0.000
	Rural	1906 (30.51)	1474 (77.33)	432 (22.67)			
	Nomadic	1954 (31.28)	1069 (54.71)	885 (45.29)			

Wealth index	Lowest	1836 (29.39)	1085 (59.10)	751 (40.90)	461.74	4	0.000
	Second	874 (13.99)	564 (64.53)	310 (35.47)			
	Middle	712 (11.40)	533 (74.86)	179 (25.14)			
	Fourth	1124 (17.99)	927 (82.47)	197 (17.53)			
	Highest	1701 (27.23)	1495 (87.89)	206 (12.11)			

**Table 4:** Multicollinearity test.

Variable	VIF	1/VIF
Wealth quantile	2.93	0.341
Region	1.06	0.944
Residence	2.61	0.383
Age in the 5-year group	1.10	0.906
Radio	1.07	0.935
TV	1.50	0.668
Education level	1.39	0.718
Mean VIF	1.67	

**Table 5:** Multivariable logistic regression analysis of knowledge and awareness of HIV/AIDS among women in Somaliland using SLDHS 2020 data.

Variable	Levels	Odds ratio (OR)	Standard error	Confidence interval (CI)	*p* value
Age in 5-year groups	15–19	Ref			
	20–24	0.55	0.05	(0.46, 0.67)	0.000
	25–29	0.40	0.04	(0.33, 0.49)	0.000
	30–34	0.37	0.04	(0.30, 0.46)	0.000
	35–39	0.33	0.04	(0.27, 0.42)	0.000
	40–44	0.34	0.05	(0.27, 0.45)	0.000
	45–49	0.34	0.06	(0.25, 0.47)	0.000

Education level	No education	Ref			
	Primary	0.47	0.05	(0.38, 0.57)	0.000
	Secondary	0.19	0.04	(0.13, 0.28)	0.000
	Higher	0.043	0.03	(0.01, 0.17)	0.000

Radio listening frequency	At least once a week	Ref			
	Less than once a week	1.47	0.58	(0.68, 3.20)	0.329
	Not at all	2.05	0.51	(1.26, 3.34)	0.004

Television watching frequency	At least once a week	Ref			
	Less than once a week	1.01	0.26	(0.61, 1.69)	0.960
	Not at all	1.29	0.19	(0.97, 1.72)	0.084

Region	Awdal	Ref			
	Marodijeh	0.41	0.05	(0.33, 0.52)	0.000
	Togdheer	0.76	0.08	(0.61, 0.93)	0.009
	Sool	1.20	0.13	(0.97, 1.48)	0.092
	Sanaag	0.75	0.08	(0.60, 0.93)	0.009

Type of place of residence	Rural	Ref			
	Urban	1.47	0.14	(1.22, 1.78)	0.000
	Nomadic	2.80	0.36	(2.18, 3.60)	0.000

Wealth index	Lowest	Ref			
	Second	0.99	0.09	(0.82, 1.20)	0.932
	Middle	1.08	0.14	(0.83, 1.40)	0.574
	Fourth	0.81	0.11	(0.63, 1.06)	0.124
	Highest	0.83	0.12	(0.62,1.09)	0.181

Cons		0.28	0.09	(0.15, 0.52)	0.000

## Data Availability

Data will be available upon request from the corresponding authors.

## References

[1] Mamtaz A. (1999). HIV/AIDS: Response to the Pandemic in Bangladesh. *Journal of Preventive and Social Medicine: JOPSOM: A Bi-Annual Journal of the National Institute of Preventive and Social Medicine*.

[2] UNAIDS (2022). UNAIDS Data 2022. *Urban Affairs Quarterly*.

[3] Who (2023). *People Living With HIV People Acquiring HIV People Dying From HIV-Related Causes*.

[4] Cabezas M. C., Fornasini M., Dardenne N., Borja T., Albert A. (2013). A Cross-Sectional Study to Assess Knowledge About HIV/AIDS Transmission and Prevention Measures in Company Workers in Ecuador. *BMC Public Health*.

[5] UNAIDS (2011). *Global Report 2012 With Annexes [Internet]*.

[6] UNAIDS_2019_core-epidemiology-slides_en.

[7] Abdool Karim Q., Sibeko S., Baxter C. (2010). Preventing HIV Infection in Women: A Global Health Imperative. *Clinical Infectious Diseases*.

[8] Quinn T. C., Overbaugh J. (2005). HIV/AIDS in Women: An Expanding Epidemic. *Science*.

[9] Bunyasi E. W., Coetzee D. J. (2017). Relationship Between Socioeconomic Status and HIV Infection: Findings From a Survey in the Free State and Western Cape Provinces of South Africa. *BMJ Open*.

[10] Gavin L., Galavotti C., Dube H. (2006). Factors Associated With HIV Infection in Adolescent Females in Zimbabwe. *Journal of Adolescent Health*.

[11] Ang L. W., Low C., Wong C. S. (2021). Epidemiological Factors Associated With Recent HIV Infection Among Newly-Diagnosed Cases in Singapore, 2013–2017. *BMC Public Health*.

[12] Pereira B. d S., Costa M. C. O., Amaral M. T. R., Costa H. S. D, Silva C. A. L. D, Sampaio V. S. (2014). Fatores Associados à Infecção Pelo HIV/AIDS Entre Adolescentes e Adultos Jovens Matriculados em Centro de Testagem e Aconselhamento no Estado da Bahia, Brasil. *Ciência & Saúde Coletiva*.

[13] World Health Organization (2020). *Health and Well-Being Profile of the Eastern Mediterranean Region: An Overview of the Health Situation in the Region and its Countries in 2019*.

[14] Morrison J., Malik S. M. M. R. (2024). Health Equity in Somalia? An Evaluation of the Progress Made From 2006 to 2019 in Reducing Inequities in Maternal and Newborn Health. *International Journal for Equity in Health*.

[15] Ali A. M., Handuleh J., Patel P. (2014). The Most Fragile State: Healthcare in Somalia. *Medicine, Conflict and Survival*.

[16] Bile K., Warsame M., Ahmed A. D. (2022). Fragile States Need Essential National Health Research: The Case of Somalia. *Lancet Global Health*.

[17] Aden J. A., Ahmed H. J., Östergren P. O. (2019). Causes and Contributing Factors of Maternal Mortality in Bosaso District of Somalia. A Retrospective Study of 30 Cases Using a Verbal Autopsy Approach. *Global Health Action*.

[18] Unaids (2024). *2024 Global AIDS Report—The Urgency of Now: AIDS at a Crossroads*.

[19] CSD Central Statistics Department (2020). *The Somaliland Health and Demographic Survey (SLHDS)*.

[20] Teshome R., Youjie W., Habte E., Kasm N. M. (2016). Comparison and Association of Comprehensive HIV/AIDS Knowledge and Attitude Towards People Living With HIV/AIDS Among Women Aged 15-49 in Three East African Countries: Burundi, Ethiopia and Kenya. *Journal of AIDS & Clinical Research*.

[21] Ankunda D. (2017). *Determinants of Comprehensive Knowledge of HIV/AIDS Among Women of the Reproductive Age (15-49) in Uganda*.

[22] World Health Organization (2015). *Global Health Observatory (GHO) Data*.

[23] Doms A., Schroeder M. (2005). GoPubMed: Exploring PubMed With the Gene Ontology. *Nucleic Acids Research*.

[24] Falagas M. E., Pitsouni E. I., Malietzis G. A., Pappas G. (2008). Comparison of PubMed, Scopus, Web of Science, and Google Scholar: Strengths and Weaknesses. *The FASEB Journal*.

[25] Wilson D. (2006). *HIV Epidemiology: A Review of Recent Trends and Lessons*.

[26] Sierra S., Kupfer B., Kaiser R. (2005). Basics of the Virology of HIV-1 and its Replication. *Journal of Clinical Virology*.

[27] Medzhitov R., Littman D. (2008). HIV Immunology Needs a New Direction. *Nature*.

[28] Flexner C. (2007). HIV Drug Development: The Next 25 Years. *Nature Reviews Drug Discovery*.

[29] Gillies P. (1996). The Contribution of Social and Behavioral Science to HIV/AIDS Prevention. *AIDS in the World II: Global Dimensions, Social Roots and Responses*.

[30] Ress H. E. (2012). *Changing the Lens of Stigma: An Exploration of Disclosure in Self-Portraits by South Africans Living With HIV in the Through Positive Eyes Arts Initiative*.

[31] Oljira L., Berhane Y., Worku A. (2013). Assessment of Comprehensive HIV/AIDS Knowledge Level Among In‐School Adolescents in Eastern Ethiopia. *Journal of the International AIDS Society*.

[32] Sohn A., Park S. (2012). HIV/AIDS Knowledge, Stigmatizing Attitudes, and Related Behaviors and Factors That Affect Stigmatizing Attitudes Against HIV/AIDS Among Korean Adolescents. *Osong Public Health and Research Perspectives*.

[33] UNAIDS (2008). *2008 Report on the Global AIDS Epidemic*.

[34] Teshale A. B., Yeshaw Y., Alem A. Z. (2022). Comprehensive Knowledge About HIV/AIDS and Associated Factors Among Women of Reproductive Age in Sub-Saharan Africa: A Multilevel Analysis Using the Most Recent Demographic and Health Survey of Each Country. *BMC Infectious Diseases*.

[35] Mude W., Oguoma V. M., Gesesew H. A. (2020). HIV/AIDS Knowledge and Attitudes Assessment Among Women of Child-Bearing Age in South Sudan: Findings From a Household Survey. *PLoS One*.

[36] Mohamud L. A., Hassan A. M., Nasir J. A. (2023). Determinants of HIV/Aids Knowledge Among Females in Somalia: Findings From 2018 to 2019 SDHS Data. *HIV*.

[37] Agegnehu C. D., Geremew B. M., Sisay M. M. (2020). Determinants of Comprehensive Knowledge of HIV/AIDS Among Reproductive Age (15–49 Years) Women in Ethiopia: Further Analysis of 2016 Ethiopian Demographic and Health Survey. *AIDS Research and Therapy*.

[38] Gebremedhin S. A., Youjie W., Tesfamariam E. H. (2017). Predictors of HIV/AIDS Knowledge and Attitude Among Young Women of Nigeria and Democratic Republic of Congo: Cross-Sectional Study. *Journal of AIDS & Clinical Research*.

[39] Goldstein M., Archary M., Adong J. (2023). Systematic Review of mHealth Interventions for Adolescent and Young Adult HIV Prevention and the Adolescent HIV Continuum of Care in Low to Middle Income Countries. *AIDS and Behavior*.

[40] Madiba S., Ngwenya N. (2017). Cultural Practices, Gender Inequality and Inconsistent Condom Use Increase Vulnerability to HIV Infection: Narratives From Married and Cohabiting Women in Rural Communities in Mpumalanga Province, South Africa. *Global Health Action*.

[41] Mohamud L. A., Hassan A. M., Nasir J. A. (2023). Determinants of HIV/Aids Knowledge Among Females in Somalia: Findings From 2018 to 2019 SDHS Data. *HIV*.

[42] Turk T. (2017). *An Integrated Approach to Strategic Communication and Condom Social an Integrated Approach to Strategic Communication and Condom Social Marketing to Address the HIV/AIDS Epidemic in Papua New Guinea*.

[43] Abdikarim H., Ali M. A., Abokor A. H. (2025). Prevalence and Determinants of Heart Disease in Somaliland: An Analysis of the 2020 Somaliland Demographic and Health Survey (SLDHS). *Current Problems in Cardiology*.

[44] Abdikarim H., Muse A. H., Hassan M. A., Muse Y. H. (2025). Prevalence and Determinants of Home Delivery Among Pregnant Women in Somaliland: Insights From SLDHS 2020 Data. *Atención Primaria*.

[45] Ismail H. M., Muse A. H., Hassan M. A., Muse Y. H., Nadarajah S. (2024). Analyzing Unimproved Drinking Water Sources and Their Determinants Using Supervised Machine Learning: Evidence From the Somaliland Demographic Health Survey 2020. *Water*.

[46] Muse A. H., Mwalili S., Ngesa O., Chesneau C., Alshanbari H. M., El-Bagoury A. A. H. (2022). Amoud Class for Hazard-Based and Odds-Based Regression Models: Application to Oncology Studies. *Axioms*.

[47] Ali M. A., Ali A. O., Abokor A. H., Farih O. A., Muse A. H. (2024). Prevalence and Determinants of Non-Communicable Diseases Among Child-Bearing Women in Somaliland From a 2020 Nationwide Survey in Somaliland. *Discover Public Health*.

[48] Muse Y. H., Hassan M. A., Abdikarim H. (2025). Relationship Between Lifestyle Factors and Cardiovascular Disease Prevalence in Somaliland: A Supervised Machine Learning Approach Using Data From Hargeisa Group Hospital, 2024. *Current Problems in Cardiology*.

[49] Smith K. L., Weir P. L., Till K., Romann M., Cobley S. (2018). Relative Age Effects Across and Within Female Sport Contexts: A Systematic Review and Meta-Analysis. *Sports Medicine*.

[50] Maheu-Giroux M., Marsh K., Doyle C. M. (2019). National HIV Testing and Diagnosis Coverage in Sub-Saharan Africa: A New Modeling Tool for Estimating the ‘First 90’from Program and Survey Data. *AIDS*.

[51] Vrancken B., Zhao B., Li X. (2020). Comparative Circulation Dynamics of the Five Main HIV Types in China. *Journal of Virology*.

[52] Gilmour B., Alene K. A., Atalell K. A., Clements A. C. A. (2023). The Prevalence of HIV Infection in Minority Indigenous Populations of the South-East Asia and Western Pacific Regions: A Systematic Review and Meta-Analysis. *AIDS and Behavior*.

[53] Brown M. J., Cohen S. A., DeShazo J. P. (2020). Psychopathology and HIV Diagnosis Among Older Adults in the United States: Disparities by Age, Sex, and Race/Ethnicity. *Aging & Mental Health*.

[54] Lin L. L., Lakomy D. S., Chiao E. Y. (2020). Clinical Trials for Treatment and Prevention of HIV-Associated Malignancies in Sub-Saharan Africa: Building Capacity and Overcoming Barriers. *JCO Global Oncology*.

[55] Nyirenda M., Mnqonywa N., Tutshana B., Naidoo J., Kowal P., Negin J. (2022). An Analysis of the Relationship Between HIV Risk Self-Perception With Sexual Behaviour and HIV Status in South African Older Adults. *African Journal of AIDS Research*.

[56] Mitiku A. K., Yirdaw B. W., Alem H., Ferede W. Y., Erega B. B. (2024). Fertility Desire and Associated Factors Among Antiretroviral Therapy Users in South Gondar Zone, Northwest Ethiopia, 2022. *SAGE Open Medicine*.

[57] Bintabara D. (2021). Addressing the Huge Poor–Rich Gap of Inequalities in Accessing Safe Childbirth Care: A First Step to Achieving Universal Maternal Health Coverage in Tanzania. *PLoS One*.

[58] Farih O. A., Ali A. O., Abokor A. H., Ali M. A., Muse A. H., Egge A. A. A. (2025). Prevalence and Factors Associated With Female Genital Mutilation Among Daughters Using Somalia Demographic Health Survey Data, SDHS 2020. *Atención Primaria*.

[59] Yeshaneh A., Lencha A., Aweke A. M. (2021). Consistent Condom Utilization and Associated Factors Among HIV Positive Clients Attending ART Clinic at Pawi General Hospital, North West Ethiopia. *PLoS One*.

